# Soil Properties, Nutrient Dynamics, and Soil Enzyme Activities Associated with Garlic Stalk Decomposition under Various Conditions

**DOI:** 10.1371/journal.pone.0050868

**Published:** 2012-11-30

**Authors:** Xu Han, Zhihui Cheng, Huanwen Meng

**Affiliations:** State Key Laboratory of Crop Stress Biology in Arid Areas and College of Horticulture, Northwest A&F University, Yangling, Shaanxi, China; Argonne National Laboratory, United States of America

## Abstract

The garlic stalk is a byproduct of garlic production and normally abandoned or burned, both of which cause environmental pollution. It is therefore appropriate to determine the conditions of efficient decomposition, and equally appropriate to determine the impact of this decomposition on soil properties. In this study, the soil properties, enzyme activities and nutrient dynamics associated with the decomposition of garlic stalk at different temperatures, concentrations and durations were investigated. Stalk decomposition significantly increased the values of soil pH and electrical conductivity. In addition, total nitrogen and organic carbon concentration were significantly increased by decomposing stalks at 40°C, with a 5∶100 ratio and for 10 or 60 days. The highest activities of sucrase, urease and alkaline phosphatase in soil were detected when stalk decomposition was performed at the lowest temperature (10°C), highest concentration (5∶100), and shortest duration (10 or 20 days). The evidence presented here suggests that garlic stalk decomposition improves the quality of soil by altering the value of soil pH and electrical conductivity and by changing nutrient dynamics and soil enzyme activity, compared to the soil decomposition without garlic stalks.

## Introduction

The three primary functions of most ecosystems are the production, accumulation, and decomposition of organic matter [1]. The nutrient cycle has an important role to play in the maintenance and organization of ecosystems; it includes both inputs and outputs [1]. Saura-Masa et al. [2] indicated that litter fall was an output of nutrients from the aerial parts of the plants and an input of nutrients to the soil. Decomposition and nutrient cycling have been found to affect soil functions [3], [4].

Plant residues are abandoned or burned as waste under traditional farming systems. Burning and disposing of plant residues and continuous tillage have both contributed to excessive soil erosion [5], [6]. Increased environmental awareness and the resultant need for efficient agricultural practices have made the use of crop residues increasingly attractive. Because the management of crop residues is a vital component of sustainable agricultural systems, it has attracted much interest in recent years as a method for increasing soil organic matter and the nutrient supply capacity of the soil and of reducing the damage caused by the burning of residue [7].

Cover crops are crops planted to prevent soil erosion and provide green manure. They are considered highly suitable for use in an integrated approach to agriculture, because they can supplement agro-ecosystems, improve soil properties, promote nutrient cycling, and facilitate pest management [8]. Cover crop decomposition is an essential part of ecosystem functioning [9]. Certain factors can regulate the decomposition rate. These include plant residue composition, soil nutrients composition, and the physical environment [10], [11]. Early research has indicated that the effects of plant residues are complicated and rely on a delicate balance between promotive and inhibitory effects [12]. In addition to determining the recognised role of crop residues as a source of nutrient [13], studies performed in natural ecosystems, laboratories, and different farming systems have indicated that the addition of plant residues and other types of organic matter to the soil may decrease plant growth and influence the activities of enzymes and the microbial populations in the soil [13]–[16].

The ecological strategies of plant species are determined by functional traits that respond to ground conditions and influence the ecosystem [17]–[19]. The study of functional traits therefore plays an important role in research into the physiology and ecology of in plant residue decomposition. Moreover, the use of various vegetative residues to amend soil can improve the soil biological environment and plant growth conditions [20], [21]. The use of auxiliary crops during the fallow period in degraded and unfertile fields can reduce nutrient loss, prevent soil erosion, improve the biological environment, and enhance crop productivity [22]–[24]. Previous studies have reported that crop residues affect soil nitrogen dynamics and ensure the availability of inorganic nitrogen in intensive production systems [22], [25]. Some early studies have also shown that the decomposed products of crop residues can promote plant growth and others can inhibit it.

Garlic (*Allium sativum* L.) is used as both a vegetable and in medicinal applications. It is not only rich in nutrients but also possesses antibacterial properties and can be used to prevent and control disease. In vegetable production, it is generally considered a good previous crop. Garlic stalks are a byproduct of garlic production, and they can be a good biological resource [26]. In practice, garlic stalks are usually abandoned or burned as waste. As a result, not only is the rate of utilization of garlic stalks very low, but environmental pollution is also produced. Using this resource efficiently and reasonably would reduce environmental pollution, change ecosystems, and foster sustainable agriculture.

**Table 1:** Basic characteristics of original soil and garlic stalk.

Samples	pHvalue	EC value(µs • cm^−1^)	Totalnitrogen(g • kg^−1^)	Total organiccarbon(g • kg^−1^)	C/N	Organicmatter(g • kg^−1^)	Totalphosphorus(g • kg^−1^)	Totalpotassium(g • kg^−1^)	Cellulosecontent(%)
Original soil	7.75	267.0	0.99	10.55	10.61	18.19	0.89	7.96	0
Garlic stalk	7.44	653.0	0.24	353.51	146.87	609.46	16.46	0.27	0.68

Previous studies on garlic stalks have mainly focused on the allelopathy of garlic straw extracts, such as ultrasonic and aqueous extracts [27], [28]. Our recent studies indicated the importance of studying crop litter decomposition under natural and near-natural conditions using a quantitative size–density method, instead of confining litter in mesh bags because these mesh bags can create a microenvironment different from the one created by placing litter in direct contact with soil [29]. The overall objective of this study was to investigate the effects of garlic stalk decomposition in soil under different conditions on soil properties, nutrient dynamics, and soil enzyme activities. In this context, different durations of decomposition, different decomposition temperatures and different ratios of garlic stalk to soil were used to adjust the decomposition conditions.

**Figure 1:**
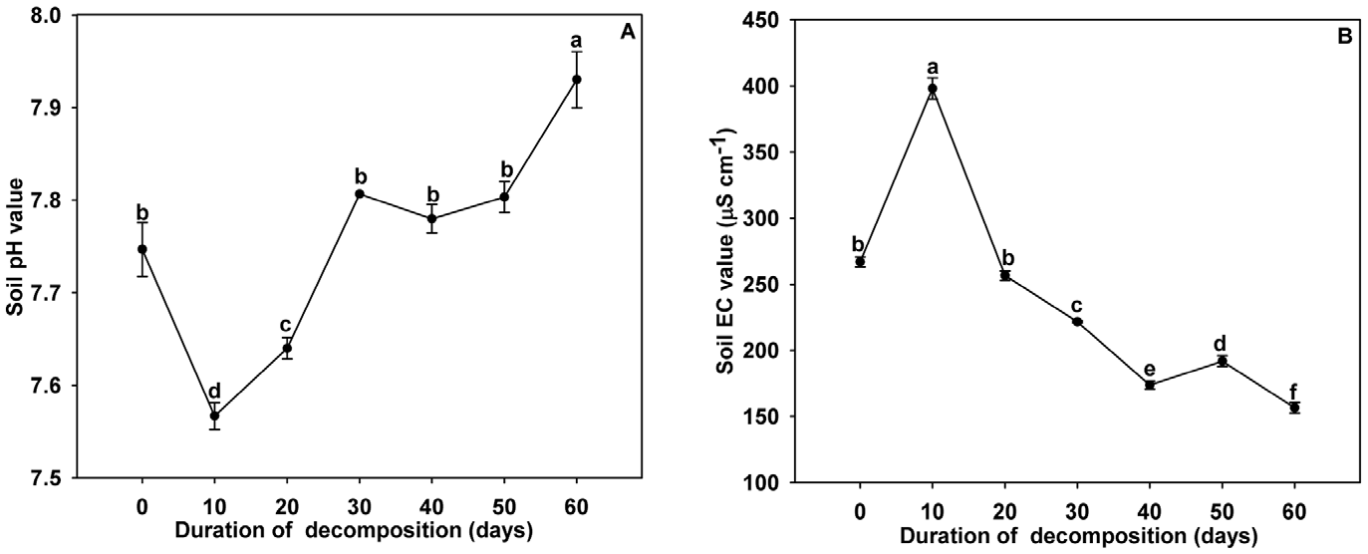
Effects of the duration of garlic stalk decomposition on soil pH (A) and EC (B). Error bars represent as the standard error of the mean. Different letters above the error bars indicate significant differences at the 0.05 level (ANOVA and Duncan’s multiple range test), n = 3.

## Materials and Methods

### Site Description

The field experiment was conducted in a plastic tunnel at the Horticulture Experimental Station (34° 16' N, 108° 4' E) of the College of Horticulture, Northwest A&F University, Yangling, Shaanxi Province, China. Garlic (cv. G064) stalks were collected from a local garlic production base field in June 2010. The harvested garlic stalks were dried under natural field conditions, ground into homogenised powder using a grinding machine, and then stored in the dark at room temperature until use. The basic characteristics of original experimental soil and garlic stalks were assayed and are shown in Table 1.

**Table 2:** Effects of different garlic stalk decomposition durations on dynamic changes in soil nutrients.

Decomposingduration(days)	Totalnitrogen(g • kg^−1^)	Totalorganic carbon(g • kg^−1^)	C/N	Organicmatter(g • kg^−1^)	Total phosphorus(g • kg^−1^)	Totalpotassium(g • kg^−1^)
0	0.99 e	10.55 bc	10.61 abc	18.19 bc	0.89 a	7.96 b
10	1.15 b	14.06 a	12.24 ab	24.23 a	0.90 a	13.74 a
20	1.12 bc	15.20 a	13.50 a	26.20 a	0.88 ab	13.98 a
30	1.01 e	12.33 ab	12.16 ab	21.25 ab	0.77 bc	13.93 a
40	1.10 c	10.33 bc	9.38 bc	17.80 bc	0.76 bc	14.15 a
50	1.06 d	8.43 c	7.93 c	14.53 c	0.70 c	14.03 a
60	1.21 a	10.61 bc	8.76 c	18.30 bc	0.85 ab	13.97 a

The data are presented as soil nutrient dynamics. Different letters in the same column indicate significant differences at the 0.05 level (ANOVA and Duncan’s multiple range test), n = 3.

**Figure 2:**
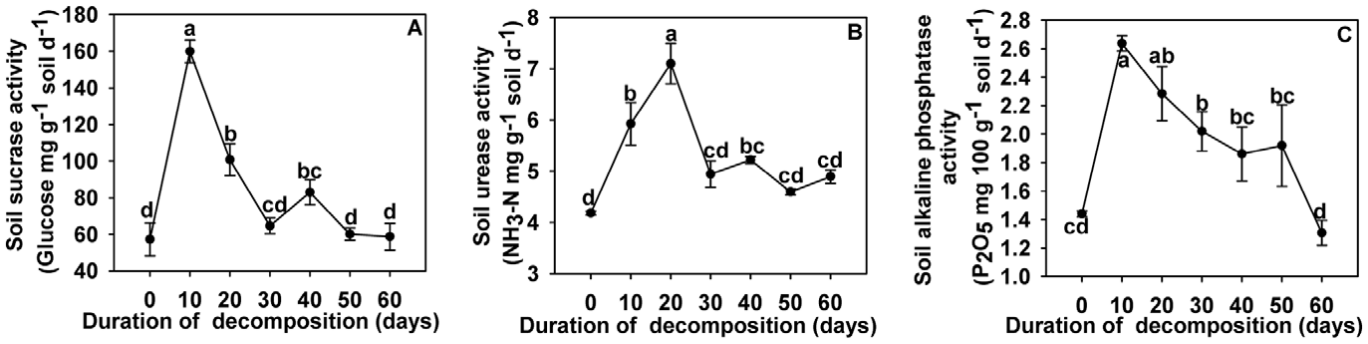
Effects of duration of garlic stalk decomposition on soil enzyme activity. Effects of different durations of garlic stalk decomposition on soil sucrase activity (A), soil urease activity (B) and soil alkaline phosphatase activity (C). Error bars represent the standard error of the mean. Different letters above the error bars indicate significant differences at the 0.05 level (ANOVA and Duncan’s multiple range test), n = 3.

### Experimental Design

#### Experiment 1 Effects of different durations of garlic stalk decomposition on soil properties, dynamic changes in nutrients, and enzyme activity

Garlic stalk powder (GSP) was mixed with soil at a ratio of 3 g dry weight per 100 g soil, which was then used to fill pots (15 cm × 15 cm × 15 cm). GSP mixed with soil was allowed to decompose naturally for 0, 10, 20, 30, 40, 50 or 60 days starting on February 15, 2011. Enough water was added to each pot to maintain the soil water content at or above 40% throughout the decomposition periods. Plastic sheets were placed under each pot in order to prevent moisture loss. Experiments were performed in triplicate and a total of 105 pots were placed in a plastic tunnel and arranged in a randomised block design. After the specified decomposition period, the soil samples from all the pots in each treatment group were homogenized, dried in a dark room, and sieved through a 1 mm or 0.149 mm sieve to determine soil properties, and soil enzyme activity, and nutrient dynamics.

**Figure 3:**
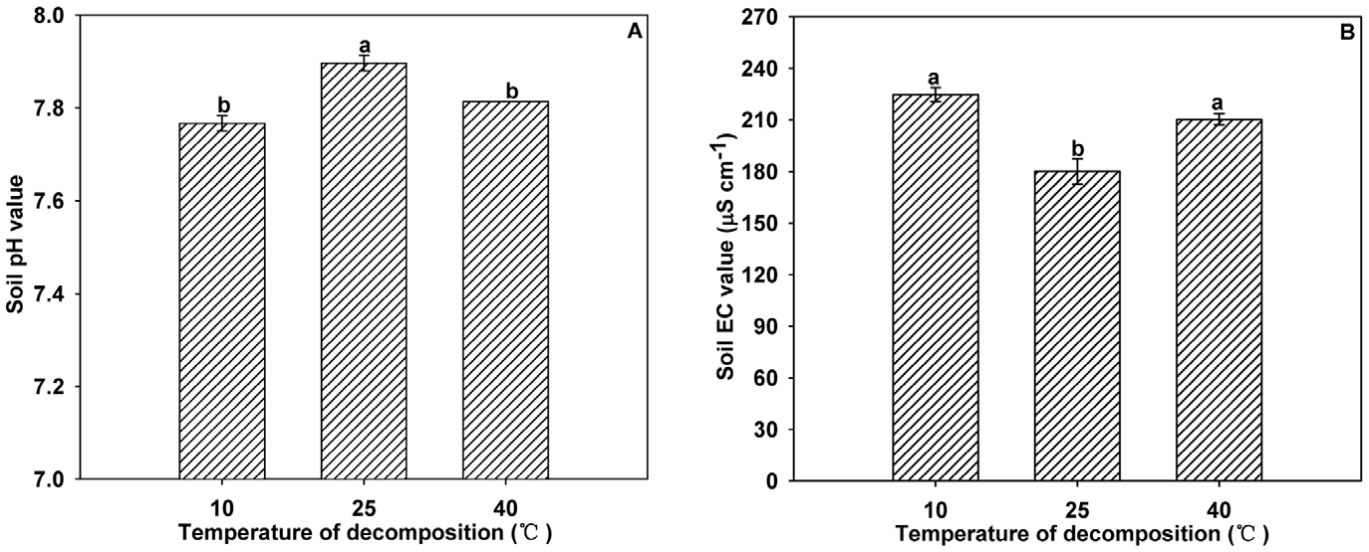
Effects of different garlic stalk decomposition temperatures on soil pH (A) and EC (B). Error bars represent the standard error of the mean. Different letters above the histograms indicate significant differences at the 0.05 level (ANOVA and Duncan’s multiple range test), n = 3.

**Table 3:** Effects of different garlic stalk decomposition temperatures on dynamic changes in soil nutrients.

Decomposing temperature(°C)	Totalnitrogen(g • kg^−1^)	Totalorganic carbon(g • kg^−1^)	C/N	Organicmatter(g • kg^−1^)	Total phosphorus(g • kg^−1^)	Totalpotassium(g • kg^−1^)
10	0.86 c	8.85 b	10.59 a	15.26 b	0.58 a	13.35 a
25	0.90 b	10.00 a	11.17 a	17.24 a	0.59 a	13.93 a
40	0.97 a	10.11 a	10.42 a	17.43 a	0.44 a	9.49 b

The data are presented as soil nutrient dynamics. Different letters in the same column indicate significant differences at the 0.05 level (ANOVA and Duncan’s multiple range test), n = 3.

#### Experiment 2 Effects of different garlic stalk decomposition temperatures on soil properties, dynamic changes in nutrients, and enzyme activity

GSP was mixed with soil at a ratio of 3 g dry weight per 100 g soil, which was then used to fill pots (15 cm × 15 cm × 15 cm). GSP mixed with soil was allowed to decompose at 10°C, 25°C and 40°C in the growth chamber (RXZ, Ningbo Jiangnan Instrument Factory, Ningbo, China) for 30 days starting on February 15, 2011. Enough water was added to each pot to keep the soil water content at least 40% throughout the decomposition period. Plastic sheets were placed under each pot to prevent moisture loss. The experiments were performed in triplicate and a total of 45 pots were arranged in a randomized block design. After the specified decomposition treatments, the soil samples from all the pots in each treatment were homogenized, dried in a dark room, and sieved through a 1 mm or 0.149 mm sieve to determine soil properties, and soil enzyme activity, and nutrient dynamics.

#### Experiment 3 Effects of different concentrations of decomposed garlic stalk on soil properties, dynamic changes in nutrients, and enzyme activity

GSP was mixed with soil at three concentrations (1, 3, or 5 g dry weight per 100 g soil) and filled in the pots (15 cm × 15 cm × 15 cm). Blank control pots were filled with the same amount of soil by weight but no garlic stalks. The experiment was performed in triplicate; a total of 60 pots were placed in a plastic tunnel and arranged in a randomised block design. Decomposition was carried out naturally for 30 days starting on February 15, 2011. Water was added to each pot to keep soil water content at least 40% throughout the decomposition periods. Plastic sheets were placed under each pot to prevent moisture loss. After the specified decomposition treatments, the soil samples from all the pots in each treatment were homogenized, dried in a dark room and sieved through a 1 mm or 0.149 mm sieve to determine soil properties, and soil enzyme activity, and nutrient dynamics.

**Figure 4:**
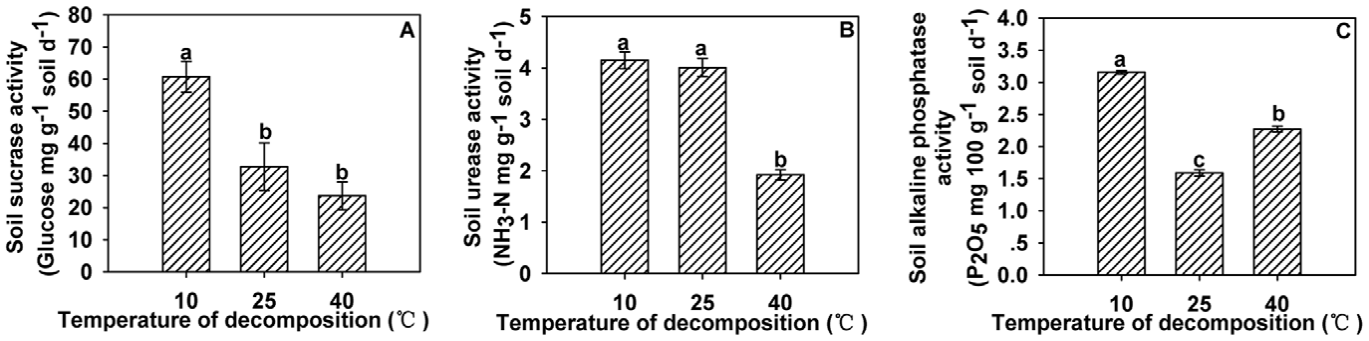
Effects of different garlic stalk decomposition temperatures on soil enzyme activities. Effect of different decomposition temperature of garlic stalk on soil sucrase activity (A), soil urease activity (B) and soil alkaline phosphatase activity (C). Error bars represent the standard error of the mean. Different letters above the histograms indicate significant differences at the 0.05 level (ANOVA and Duncan’s multiple range test), n = 3.

**Figure 5:**
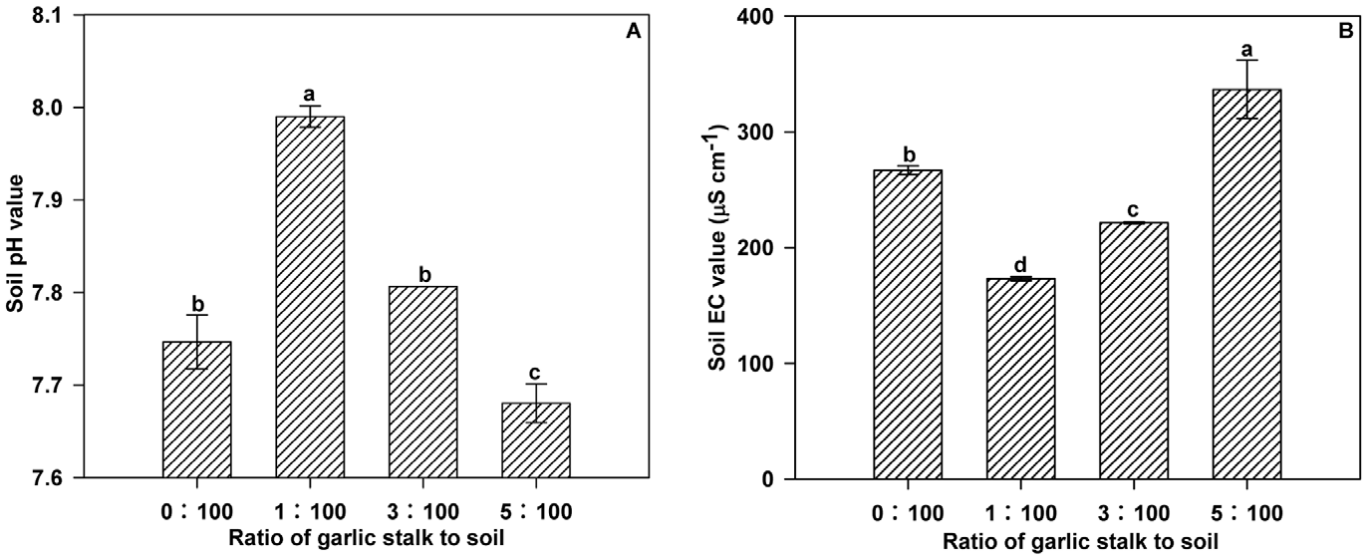
Effects of different concentrations of decomposed garlic stalk on soil pH (A) and EC (B). Error bars represent the standard error of the mean. Different letters above the error bars indicate significant differences at the 0.05 level (ANOVA and Duncan’s multiple range test), n = 3.

**Table 4:** Effects of different concentrations of decomposed garlic stalk on dynamic changes in soil nutrients.

Ratio of garlic stalk to soil	Totalnitrogen(g • kg^−1^)	Totalorganic carbon(g • kg^−1^)	C/N	Organicmatter(g • kg^−1^)	Total phosphorus(g • kg^−1^)	Totalpotassium(g • kg^−1^)
0 ∶ 100	0.99 b	10.55 b	10.61 a	18.19 b	0.89 a	7.96 b
1 ∶ 100	0.94 c	10.45 b	11.10 a	18.02 b	0.82 ab	13.18 a
3 ∶ 100	1.01 b	12.37 b	12.20 a	21.32 b	0.77 b	13.93 a
5 ∶ 100	1.21 a	15.09 a	12.44 a	26.01 a	0.66 c	13.11 a

The data are presented as the soil nutrient dynamics. Different letters in the same column indicate significant differences at the 0.05 level (ANOVA and Duncan’s multiple range test), n = 3.

**Figure 6:**
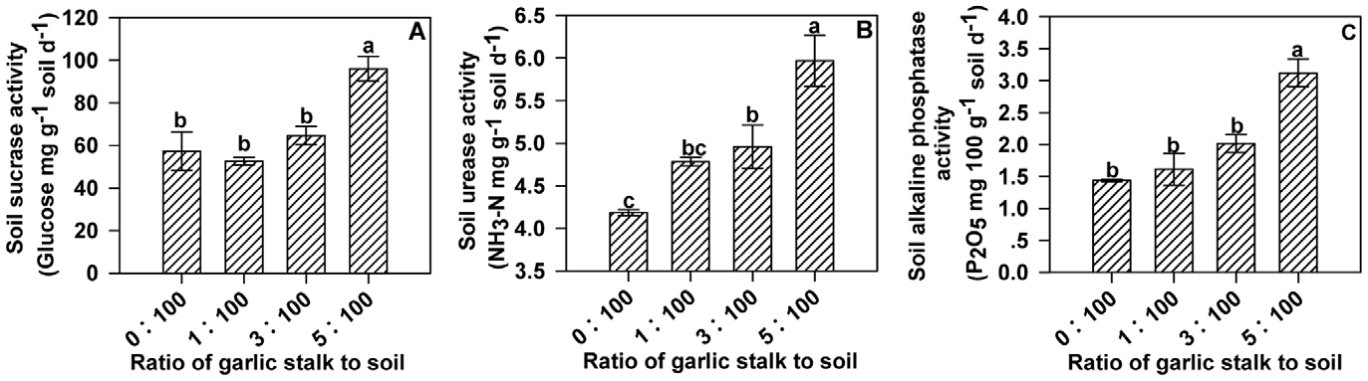
Effects of different concentrations of decomposed garlic stalk on soil enzyme activities. Effects of different concentrations of decomposed garlic stalk on soil sucrase activity (A), soil urease activity (B) and soil alkaline phosphatase activity (C). Error bars represent the standard error of the mean. Different letters above the error bars indicate significant differences at the 0.05 level (ANOVA and Duncan’s multiple range test), n = 3.

### Determination of Soil Properties

Soil samples were extracted with distilled water (1∶1 water to soil suspension) and analyzed for soil pH value by a pH meter (PHS-3C, LIDA, Shanghai, China) [30]. Soil electrical conductivity (EC) value (5∶1 distilled water to soil suspension) was determined using a microprocessor conductivity meter (DDS-12DW, Xiaoshan, China) [30].

### Determination of Nutrient Dynamics

Total nitrogen (N) content was analyzed by boiling away concentrated sulfuric acid boiled using an Automatic Kieldahl Apparatus (KDY-9830, Beijing, China) [30]. Total organic carbon (C) content was assayed using the potassium dichromate method [30].For determination the contents of total phosphorus (P) and potassium (K), the soil was wet-digested with a mixture HNO_3_–HCl–HClO_4_ (1∶3:1). Total P in the wet digestion was measured colorimetrically using the molybdenum antimony resistance method and a spectrophotometer at 880 nm, and total K was measured using an atomic absorption spectrophotometer (Z-2000, Hitachi, Japan) [30].

### Determination of Soil Enzyme Activities

The activity levels of sucrase, urease and alkaline phosphatase in soil were assayed on the basis of the release and quantitative determination of the products of glucose, NH_3_-N, and P_2_O_5_. Soil samples were incubated with an 8% sucrose solution, a 10% urea solution, and a 0.5% disodium phenyl phosphate solution in a suitable buffer solution for 24 hours at 37°C and spectrophotometric measurements were performed at 508 nm, 578 nm and 660 nm [31].

### Statistical Analysis

The data were assessed using one-way analysis of variance (ANOVA) with the SPSS 17.0 software package (SPSS Inc., Chicago, U.S.). Mean separations were performed using Duncan’s multiple range tests. Differences at *P*  = 0.05 were considered significant.

## Results

### Soil Properties, Dynamic Changes in Nutrients, and Enzyme Activities Associated with Different Durations of Garlic Stalk Decomposition

Treatments of longer durations (60 days) were associated with significantly higher soil pH values. However, significantly lower soil pH values were associated with 10 days of garlic stalk decomposition (Fig 1A). Soil EC values varied significantly with the duration of garlic stalk decomposition (Fig 1B). The highest soil EC value was associated with garlic stalk decomposition lasting 10 days, and the lowest value was associated with garlic stalk decomposition lasting 60 days.

The test results of soil nutrient dynamic changes associated with different decomposition durations are summarized in Table 2. The shorter durations (10 and 20 days) were associated with significantly higher levels of total organic carbon and organic matter. The highest total nitrogen content was observed in soil in which garlic stalks had decomposed for 60 days. However, the highest C/N ratio was associated with 20 days of garlic stalk decomposition. Total phosphorus content was significantly higher in groups subjected to 0 and 10 days of garlic stalk decomposition. Total potassium content was significantly higher after 10 to 60 days of decomposition than after 0 days of decomposition.

Enzyme activities in soil in which garlic stalks had decomposed for different periods of time are given in Fig. 2. Garlic stalk decomposition 10 days was associated with significantly higher levels of sucrase and alkaline phosphatase activity (Fig 2A and 2C). The highest urease activity was recorded in the soil in which garlic stalks had been allowed to decompose for 20 days (Fig 2B).

### Soil Properties, Dynamic Changes in Nutrients, and Enzyme Activities, Associated with Different Garlic Stalk Decomposition Temperatures

The highest soil pH values were associated with garlic stalk decomposition at 25°C, but no significant difference was observed between the decomposition at 10°C and at 40°C (Fig 3A). Garlic stalk decomposition at 25°C was associated with significant lower soil EC values than decomposition at 10°C or 40°C. No significant difference was observed between decomposition at 10°C and at 40°C (Fig 3B).

The effects of different garlic stalk decomposition temperatures on the dynamic changes in soil nutrients were assessed (Table 3). Total nitrogen content increased as decomposition temperature increased, and the highest value was observed at 40°C of decomposition. Garlic stalk decomposition at 25°C and 40°C was found to significantly promote total organic carbon and organic matter content over values observed at 10°C. However, the C/N ratio and total phosphorus content remained relatively constant across all three treatments groups. Total potassium content was significantly inhibited at 40°C but no significant difference was observed between decomposition at 10°C and at 25°C.

Garlic stalk decomposition at 10°C was associated with significantly higher sucrase activity than decomposition 25°C or 40°C, but there were no significant difference between decomposition at 25°C and at 40°C (Fig 4A). Garlic stalk decomposition at 10°C significantly promoted urease activity. However, urease activity was significantly inhibited by decomposition at 25°C (Fig 4B). The lowest alkaline phosphatase activity was associated with decomposition at 40°C, and the higher values were associated with decomposition at 10°C and 25°C (Fig 4C).

### Soil Properties, Dynamic Changes of Nutrients, and Enzyme Activities Associated with Different Concentrations of Decomposed Garlic Stalk

Soil pH (Fig 5A) and EC values (Fig 5B) varied significantly with the concentration of decomposed garlic stalk. The lowest non-zero concentration (1∶100) of decomposed garlic stalk was associated with the highest soil pH value and lowest soil EC value. These reverse was true of the highest concentration (5∶100).

Effects of different concentrations of decomposed garlic stalk on dynamic changes in soil nutrients are presented in Table 4. The highest concentrations of garlic stalk to soil (5∶100) significantly increased the concentration of total nitrogen, total organic carbon and organic matter relative to the control (0∶100). However, the C/N ratio did not differ across the treatment groups. The total phosphorus content decreased as the ratio of garlic stalk to soil increased. Total soil potassium content of all treatments was significantly higher than control.

The effects of decomposed garlic stalks on the activity levels of sucrase (Fig 6A), urease (Fig 6B) and alkaline phosphatase (Fig 6C) varied with the ratio of garlic stalk to soil. The highest activities of all three soil enzymes were observed at the highest ratio (5∶100).

## Discussion

The decomposition of plant residues is one of the important processes in the nutrient cycle of almost any ecosystem [32]. Decomposition dynamics are impacted by several main factors, such as the duration of decomposition, environmental conditions (especially temperature), the quantity of the decomposers, and the quality of plant residues as the substrate for the decomposers [33], [34]. For these reasons, three different sets of decomposition conditions, such as duration, temperature and concentrations of garlic stalks were chosen for this investigation of the effects of decomposed garlic stalks on soil properties, nutrient dynamics, and soil enzyme activities.

Soil pH and EC value are the primary factors indicating the chemical properties of the soil. The burning of plant residues and continuous cultivation of the same plant could lead to soil acidification or salinization. Soil pH can also affect nutrient availability, such as soil ion exchange capacity, nutrient adsorption and solubility, soil microbial activities, and the root uptake process [35]–[37]. Several studies have reported that high soil pH is usually associated with low soil nutrient availability [38]–[42]. In the current study, the highest soil pH value and total nitrogen content were observed when garlic stalks decomposed over 60 days. Decomposition times of 10 days, temperatures of 10°C and 40°C, and garlic stalk to soil ratios of 5∶100 were associated with significantly higher soil EC values.

Plant residue decomposition is a vital pathway in the carbon cycle and nutrient regeneration [43], [44]. Plant residue is a major source of carbon for microbes in the soil and a major route of input of soil organic carbon [45], [46]. In this way, plant residue can regulate essential biological functions in soil ecosystems. Our results showed that garlic stalk decomposition lasting 10 days or 20 days significantly increased soil total organic carbon and organic matter. These results are similar to those of one previous study that showed that many elements in plant materials were released faster after the plant materials were placed in the soil and decomposed immediately [47]. The highest values of soil total organic carbon and organic matter were observed at decomposition temperatures of 25°C and 40°C. This was consistent with the results of other studies, which reported that higher temperatures were associated with faster nutrient cycling and that air temperature could impact the degree of decomposition [48], [49]. In our study, the highest levels of total nitrogen were observed in soil where garlic stalks had decomposed at 40°C, for 60 days and concentrations of 5∶100. Several studies have reported that crops grown on legume residues experienced increased nitrogen uptake [50], [51]. In the present study, a 5∶100 stalk-to-soil ratio also significantly increased the level of total soil organic carbon and organic matter.

The activity of soil enzymes plays a key role in many ecosystem and soil management processes, and it may influence soil quality and function [52], [53]. It is usually higher in plant residues vicinity than in bulk soil. In this study, the peak sucrase and alkaline phosphatase activity was observed in soil where garlic stalks had decomposed for 10 days and peak urease activity was observed where garlic stalks had decomposed for 20 days. Soil urease activity was found to be highly responsive to nutritional conditions [54]. Dilly *et al.* [55] reported that the highest activity of urease was observed at later stages of decomposition. However, our results were not consistent with this. The results of our study showed that garlic stalk decomposition at 10°C significantly increased soil sucrase and urease activities and stalk decomposition at 10°C and 25°C significantly promoted soil alkaline phosphatase activity. These results are consistent with the previous studies stating that bogs, fens, and other wetlands exhibited less soil phosphatase activity because of the cool temperatures [56]. The highest activities of these three soil enzymes detected in this study were observed at garlic-stalk-to-soil ratios of 5∶100.

### Conclusions

The results of this study showed that different garlic stalk decomposition conditions can have a significant impact on soil properties, such as pH and EC value. Soil nutrient dynamics were also found to vary with garlic stalk decomposing conditions, such as the duration of decomposition, temperature, and the ratio of garlic stalk to soil. The highest soil enzyme activity appeared at shorter durations (10 days or 20 days), lower temperatures (10°C), and higher concentrations of stalk (5∶100).
